# Severe hypocalcemia after denosumab treatment leading to refractory ventricular tachycardia and veno-arterial extracorporeal membrane oxygenation support: a case report

**DOI:** 10.1186/s12245-023-00529-6

**Published:** 2023-08-28

**Authors:** Fumito Okuno, Asami Ito-Masui, Atsuya Hane, Keiko Maeyama, Kaoru Ikejiri, Ken Ishikura, Masashi Yanagisawa, Kaoru Dohi, Kei Suzuki

**Affiliations:** 1 Emergency and Critical Care Center, Mie University Hospital, 2-174 Edobashi, Tsu-City, Mie 514-8507 Japan; 2 Department of Hematology and Oncology, Mie University Graduate School of Medicine, 2-174 Edobashi, Tsu-City, Mie 514-8507 Japan; 3 Department of Cardiology and Nephrology, Mie University Graduate School of Medicine, 2-174 Edobashi, Tsu-City, Mie 514-8507 Japan

**Keywords:** Hypocalcemia, Ventricular tachycardia, Denosumab, Veno-arterial extracorporeal membrane oxygenation, Intra-aortic balloon pump

## Abstract

**Background:**

Severe hypocalcemia may lead to life-threatening arrhythmias. Denosumab is an effective treatment for osteoporosis that allows long intervals between doses. However, there is a risk of hypocalcemia in some patients. Due to the long half-life of denosumab, emergency physicians caring for patients presenting with symptoms of hypocalcemia may not be aware of the medication, and adverse effects may last longer.

**Case presentation:**

A 55-year-old woman with a history of systemic lupus erythematosus (SLE) and anxiety disorder called for an ambulance for symptoms of hyperventilation and muscle cramps. After evaluation at the local hospital, she developed pulseless ventricular tachycardia and was resuscitated by defibrillation by the hospital staff. After conversion to sinus rhythm, she was transported to a tertiary center. Upon arrival, pulseless ventricular tachycardia occurred again, and veno-arterial extracorporeal membrane oxygenation (ECMO) and intra-aortic balloon pumping (IABP) were implemented. Laboratory results showed severe hypocalcemia (corrected calcium level of 5.3 mg/dL) whereupon intravenous calcium supplementation was started. She had received the first dose of denosumab (60 mg) by subcutaneous injection 24 days prior to hospitalization. She was eventually weaned from ECMO and IABP support.

**Conclusion:**

Cardiac arrest due to hypocalcemia is relatively rare but can be fatal. In the present case, hyperventilation may have acutely exacerbated pre-existing hypocalcemia, leading to ventricular tachycardia. The patient had a slightly decreased serum calcium level prior to denosumab. Close monitoring may be preferable after the primary dose of denosumab in selected patients. Emergency physicians caring for patients who may be suffering from symptoms/signs of hypocalcemia must be mindful of medications that have long half-lives and affect electrolyte balance when treating fatal arrhythmia due to hypocalcemia.

## Background

Severe hypocalcemia may cause fatal arrhythmias, such as Torsade de Pointes, ventricular tachycardia, and ventricular fibrillation. In general, severe hypocalcemia (total serum concentration < 8.5 mg/dL) is considered an emergency due to the potential risk of life-threatening arrhythmia. However, as these life-threatening arrhythmias are relatively rare, the occurrence rate of such complications is not well known. Some studies imply that arrhythmia due to hypocalcemia may be a result of multiple secondary factors [1].

Denosumab is a fully human monoclonal antibody that inhibits receptor activator of nuclear factor-kappa B ligand (RANKL), an essential mediator of osteoclast formation [2]. It is used as a treatment for osteoporosis, rheumatoid arthritis, multiple myeloma, and bone metastasis. The standard dosing for osteoporosis is a subcutaneous injection of 60 mg twice yearly. To some, this is considered preferable over bisphosphonates, where compliance is a major concern [3]. A meta-analysis showed that denosumab increased bone mineral density compared to bisphosphonates [4]. The bone mineral density change from baseline at month 12 was significantly greater with denosumab compared with zoledronic acid at the lumbar spine (primary endpoint; 3.2% vs 1.1%; *P* < 0.0001), total hip (1.9% vs 0.6%; *P* < 0.0001), femoral neck (1.2% vs − 0.1%; *P* < 0.0001), and one-third radius (0.6% vs 0.0%; *P* < 0.05) [5]. Hypocalcemia is a known adverse effect of denosumab. In prospective clinical trials, hypocalcemia occurred in 2–13% of patients on denosumab, depending on the patient’s characteristics [6, 7]. However, a retrospective study showed a higher rate of hypocalcemia, affecting 35% of patients within 30 days of denosumab administration, with acute kidney insufficiency as a risk factor [8].

Severe hypocalcemia leading to life-threatening arrhythmias is rare but can be fatal. We herein report a case of refractory ventricular fibrillation associated with hypocalcemia after a single injection of denosumab that was successfully treated with aggressive intravenous calcium supplementation under veno-arterial extracorporeal membrane oxygenation (VA-ECMO) and intra-aortic balloon pumping (IABP) support.

## Case presentation

A 55-year-old woman with a medical history of systemic lupus erythematosus (SLE), interstitial pneumonia, osteoporosis, myocardial ischemia, chronic heart failure, and anxiety disorder was transported to the emergency department (ED) of a rural local hospital by ambulance with a complaint of hyperventilation and leg cramps. She had begun experiencing weakness in her upper extremities the day before. The patient was taking prednisone for SLE, and she had recently received her first subcutaneous injection of denosumab (60 mg) 24 days prior to hospitalization for her osteoporosis. Past medical records showed that she had a percutaneous intervention with stent placement in the right coronary artery 5 years prior to hospitalization, and her ejection fraction was 35–40% 2 years prior to hospitalization.

Soon after primary evaluation at the ED, the patient’s respiratory condition worsened, and she went into cardiac arrest. The electrocardiographic monitor showed ventricular tachycardia, and the patient was found to be pulseless. After cardiopulmonary resuscitation with defibrillation, the patient returned to sinus rhythm. The patient was then rapidly transferred to a tertiary-care emergency hospital without intubation for further evaluation and treatment.

**Fig. 1:**
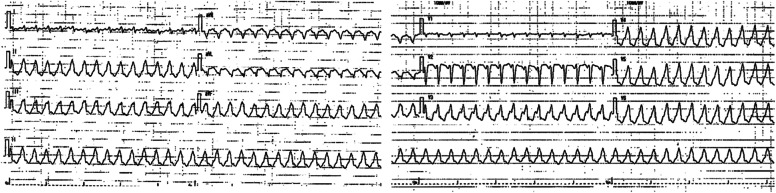
The electrocardiogram upon arrival showed ventricular tachycardia

On arrival, the patient was agitated with a blood pressure of 66/53 mmHg and she was found to have ventricular tachycardia of 151 beats/min (Fig. 1). Synchronized cardioversion was performed at 150 and 200 J with no conversion to sinus rhythm. Intravenous magnesium and lidocaine were also administered. Blood tests showed significant hypocalcemia with a corrected calcium level of 5.3 mg/dL. As the patient was transported to the catheterization laboratory for coronary angiography, she went into pulseless ventricular tachycardia. VA-ECMO was implemented with a blood flow of 4.4 L/min, sweep gas oxygen saturation of 100%, and gas flow of 3.0 L/min.

There were no features of coronary stenosis on angiography, leading to a high suspicion of hypocalcemia-induced refractory ventricular tachycardia. Other laboratory results were as follows: magnesium 1.5 mg/dL, phosphate 3.8 mg/dL, alkaline phosphatase 84 U/L, creatinine 0.8 mg/dL, vitamin D 10.9 pg/dl, parathyroid hormone 67 pg/dL, and 25(OH) vitamin D 68 pg/mL. Computed tomography was performed after the implementation of VA-ECMO, revealing new bilateral pulmonary infiltration.

Intravenous supplementation of calcium chloride, magnesium, and phosphate was initiated. For the treatment of hypocalcemia, calcium chloride was started since this treatment was readily available in the ED. After a careful evaluation of the patient’s medical history, it became clear that the patient had received her first injection of 60 mg of denosumab 24 days prior to hospitalization for osteoporosis treatment. In addition, the serum calcium level prior to denosumab treatment had been slightly low at 7.9 mg/dL. We therefore suspected that denosumab might have caused hypocalcemia, which was acutely exacerbated due to hyperventilation. The patient also had chronic diarrhea, which may have caused hypomagnesemia and hypocalcemia. Other diseases that cause hypocalcemia, such as vitamin D deficiency and hypoparathyroidism, were excluded based on laboratory tests.

**Fig. 2:**
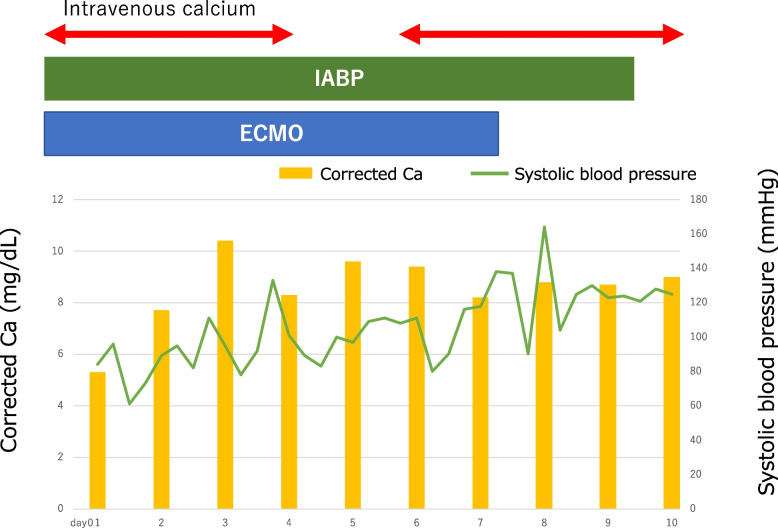
The clinical course of the patient after arrival

The following day, IABP was initiated due to a low ejection fraction and poor systemic perfusion. Her serum calcium level reached a normal level by the second day, but high-dose intravenous supplementation was continued for 10 days, followed by maintenance intravenous supplementation for 3 weeks. The patient was weaned from ECMO on the 7th day (Fig. 2) and remained in the intensive care unit for 23 days before being transferred for rehabilitation after 78 days.

## Discussion and conclusions

We encountered a case of severe hypocalcemia leading to ventricular tachycardia after the initial injection of denosumab. Clinically, hypocalcemia may present with Chvostek’s sign, Trousseau’s sign, tetany, peripheral and extremity paresthesia, muscle cramps, laryngospasm, bronchospasm, confusion, and seizures. Hypocalcemia-induced cardiac arrest is a rare yet fatal condition. Several factors in the present case, such as the prior injection of denosumab, chronic diarrhea, and hyperventilation might have increased the risk of severe hypocalcemia.

In the present case, the combination of VA-ECMO and IABP was used for hemodynamic support. The combination of VA-ECMO and IABP demonstrates superior efficacy in the treatment of refractory cardiogenic shock compared to VA-ECMO only [9]. This is attributed to the anticipated reduction in left ventricular afterload and augmented coronary perfusion facilitated by the integration of IABP, thereby utilizing the oxygenated blood from the ECMO circuit.

Denosumab is an effective treatment that improves bone marrow density. Changes in bone marrow density are significantly greater than with bisphosphates [10], but rates of hypocalcemia are also higher. Retrospective multivariate analyses have shown that lower baseline serum calcium levels, higher serum alkaline phosphatase elevation, chronic kidney disease, male sex, and administration of certain drugs (cytotoxic agents, vonoprazon, dexamethasone) are risk factors for denosumab-induced hypocalcemia [11–14]. In the present case, laboratory tests prior to denosumab showed serum calcium of 8.0 mg/dL, alkaline phosphatase of 200 U/L, and creatinine of 0.3 mg/dL. In addition, the patient’s low weight may have increased the risk of toxicity with denosumab. However, several pharmacokinetic studies have demonstrated that dose adjustment based on weight is not warranted [15, 16].

Denosumab has a long half-life. In healthy volunteers, the mean terminal half-life of denosumab 60 mg was reportedly 15 days, with serum concentrations peaking at around day 10 [17]. Due to this long half-life, severe hypocalcemia associated with denosumab has been reported as refractory hypocalcemia despite supplementation of calcium gluconate, requiring long periods of treatment for several weeks [18–20]. However, in the present case, the serum level of calcium increased within 48 h after intense supplementation of intravenous calcium gluconate. This suggests that hyperventilation may have been accountable for inducing acute hypocalcemia, with denosumab as an underlying factor.

Cardiac events as a result of denosumab-associated hypocalcemia are rarely reported in the literature. There have been reports of severe hypocalcemia associated with a prolonged QT interval as well as acute left heart failure after denosumab treatment [21, 22]. However, most case reports on denosumab-associated hypocalcemia present with numbness, fatigue, and weakness. Again, this may be because hyperventilation triggered an acute decrease in ionized calcium, leading to ventricular arrhythmia. We assume that she may have been hyperventilating as a symptom of anxiety disorder, although the reason is unclear. It is known that hyperventilation induces a state of respiratory alkalosis, in which the binding of ionized calcium to albumin results in a reduction of ionized calcium concentration. Unfortunately, we do not have the blood gas analysis upon arrival at the primary hospital to support this hypothesis. Acute loss of calcium may be a cause of ventricular fibrillation in hemofiltration patients [23]. Some physicians have even proposed a different treatment algorithm for acute and chronic hypocalcemia [24]. The acute-on-chronic nature of the present case may have caused life-threatening arrhythmia. That being said, it should be noted that some studies argue that cardiac arrest due to hypocalcemia may be attributed to other causes, such as co-morbidities and other electrolyte disorders [1]. In the present case, the patient had a history of myocardial ischemia and chronic heart failure with low ejection fraction, which may have contributed to refractory ventricular tachycardia. The association of severe hypocalcemia and immediately life-threatening cardiac arrhythmias in clinical practice warrants further investigation.

In conclusion, this report highlights the importance of monitoring calcium levels closely in selected patients on denosumab. Emergency care providers must be aware of symptoms and signs, life-threatening complications, and risk factors of hypocalcemia.

## Data Availability

Not applicable.

## Abbreviations

SLE: Systemic lupus erythematosus
ED: Emergency department
VA-ECMO: Veno-arterial extracorporeal membrane oxygenation
IABP: Intra-aortic balloon pumping
